# Effects of Probiotic *Bacillus* as an Alternative of Antibiotics on Digestive Enzymes Activity and Intestinal Integrity of Piglets

**DOI:** 10.3389/fmicb.2018.02427

**Published:** 2018-10-22

**Authors:** Shenglan Hu, Xuefang Cao, Yanping Wu, Xiaoqiang Mei, Han Xu, Yang Wang, Xiaoping Zhang, Li Gong, Weifen Li

**Affiliations:** ^1^Key Laboratory of Molecular Animal Nutrition and Feed Sciences, College of Animal Science, Zhejiang University, Hangzhou, China; ^2^State Key Laboratory of Livestock and Poultry Breeding, Key Laboratory of Animal Nutrition and Feed Science in South China, Ministry of Agriculture, Institute of Animal Science, Guangdong Academy of Agricultural Sciences, Guangzhou, China; ^3^Key Laboratory of Resources and Utilization of Bamboo of State Forestry Administration, China National Bamboo Research Center, Hangzhou, China

**Keywords:** piglets, antibiotics, *Bacillus amyloliquefaciens*, digestive enzyme activity, intestinal integrity

## Abstract

The previous study in our team found that supplementation of probiotic *Bacillus amyloliquefaciens* (Ba) instead of antibiotics promote growth performance of piglets. Hence, the present study was carried out to further demonstrate the effect of Ba replacement of antibiotics on digestive and absorption enzyme activity and intestinal microbiota population of piglets. A total of 90 piglets were selected and divided into three groups: G1 group was fed with basal diet supplemented with 150 mg/Kg aureomycin, G2 group was fed with 1 × 10^8^ cfu/Kg Ba and half dose of aureomycin, G3 group was used the diet with 2 × 10^8^cfu/Kg Ba replaced aureomycin. Each treatment had three replications of 10 pigs per pen. Results indicated that Ba replacement significantly increased the activities of amylase, disaccharides and Na^+^/K^+^-ATPase. And chymotrypsin activity in different section of intestine was dramatically enhanced in half replacement of aureomycin with Ba. Moreover, Ba replacement maintained the intestinal integrity with the significantly decreased activity of DAO compared with aureomycin group. Besides, supplementation with Ba increased the β-diversity of intestinal microbiota. Taken together, the current study indicated that diet supplementation with Ba instead of aureomycin increased the growth performance of piglets by improving the digestive and absorb enzyme activities, enhancing the intestinal integrity and regulating the population of intestinal micrbiota.

## Introduction

Antibiotics have long been used to promote the growth and health of piglets (van den Bogaard and Stobberingh, 2000). However, with the increasing phenomena of bacterial resistance and antibiotic residues in animal products, the use of antibiotics in feed industry has been prohibited in many nations, such as Europe, United States, Korea, and so on (Martin et al., 2015; Walsh and Wu, 2016). Therefore, many alternatives to antibiotics have been developed (Wang et al., 2012, 2017). It is well known that probiotics are an alternative strategy to antibiotics. Previous researches illustrated that probiotics enhance growth performance of poultry and swine (Wang et al., 2012), modulate immune system (González-Ortiz et al., 2013), and promote intestinal health (Tojo et al., 2014). Probiotics, with the definition of live micro-organisms, are considered to have potential benefits on the host health (Hickey et al., 2012; Djurasevic et al., 2017). Many researches demonstrated that probiotics improve growth performance (Giang et al., 2010), modulate host immunity (Deng et al., 2013), and decrease the diarrhea rate of weaned piglets (González-Ortiz et al., 2013). *Bacillus amyloliquefaciens* is one of probiotic strains, which produces a variety of commercially important enzymes to improve digestibility and absorption of nutrients (Farhadi et al., 2003; Lee et al., 2008). Recent studies of our research group have found that replacing aureomycin with *B. amyloliquefaciens* SC06 significantly improve the daily weight gain of piglets, increase antioxidant capacity (Wang et al., 2017) and decrease bacterial translocation (Ji et al., 2013). However, little information about effects of *B. amyloliquefaciens* SC06 replacement on digestibility and absorption of nutrients in piglets was found. Therefore, the aim of this study was to clarify effects of probiotic *Bacillus amyloliquefaciens* as an alternative of antibiotics on main digestive and absorb enzymes in piglet intestine.

## Materials and Methods

The experimental procedures used in the present study were approved by the Animal Care and Use Committee of Zhejiang University, and strictly followed the guidelines of the Guide for the Care and Use of Agricultural Animals in Research and Teaching.

### Animals and Experimental Treatments

A total of 90 male Duroc × Landrace × Yorkshire piglets at 42 days old were blocked by BW (average 14.57 ± 0.25 kg), and randomly divided into three groups with 10 piglets pre pan and 3 pans pre group. The three groups were (1) Group1 (G1) fed the basal diet supplemented with 150 mg/Kg aureomycin, (2) Group 2 (G2) fed the basal diet supplemented with 75 mg/Kg aureomycin and 1 × 10^8^ cfu/Kg Ba, and (3) Group 3 (G3) fed the basal diet with 2 × 10^8^ cfu/Kg Ba. The composition of the basal diet was shown in Table 1. The experimental period was 28 days. Piglets were housed in a temperature-controlled nursery and had *ad libitum* access to feed and water. Ingredient and chemical composition of the basal diet were listed in Table 1.

**Table 1:** Ingredient and chemical composition of the basal diet (as-fed basis).

Ingredient	Content (%)	Nutition levels	Content (%)
Corn	61.25	CP	19.00
soybean meal	15.79	DE (MJ/Kg)	14.11
Extruded-soybean	10.00	Ca	0.80
Imported fish meal	5.00	TP	0.63
Wheat bran	3.00	AP	0.40
Soybean oil	1.74	Lys	1.15
Premix^a^	1.00	Met + Cys	0.67
Limestone	0.98	Thr	0.77
CaHPO_4_	0.78	Trp	0.22
Salt	0.37		
Lysine-HCl	0.09		
Total	100.00		


*^a^Premix supplied per kg: 8255 IU of vitamin A, 2000 IU of vitamin D_3_, 40 IU of vitamin E, 3 mg of vitamin K_3_, 2 mg of vitamin B_1_, 4 mg of vitamin B_2_, 10 mg of vitamin B_6_, 0.05 mg of B_12_, 3 mg of vitamin PP, 15 mg of pantothenic acid, 2 mg of folic acid, 0.2 mg of biptin, 800 mg of choline chloride, 100 mg of vitamin C, 100 mg of Fe (FeSO_4_), 20 mg of Cu (CuSO_4_), 55 mg of Mn (MnO), 50 mg of Zn (ZnO), 100 mg of I (KI), 2 mg of Se (Na_2_SeO_3_)*, *and 2 mg of Co (CoSO_4_).*

### Bacterial Strain and Aureomycin

*Bacillus amyloliquefaciens* cells (China Center For Type Culture Collection, No: M2012280) (1 × 10^8^ cfu/g) were prepared by the Laboratory of Microbiology, Institute of Feed Sciences, Zhejiang University, China. Starch was used to dilute Ba and the same amount of starch was also added to each group to compensate for the difference in nutrient composition of the diets. Aureomycin was obtained from Tongyi feed agriculture and animal husbandry Co., Ltd. (Qingdao, China).

### Sample Collection

Six pigs were randomly selected from each group for sample collecting at the end of the experiment. After the slaughter, the gastrointestinal tract was immediately removed. The segments of jejunum were removed and rinsed with sterilized saline, and then the Jejunal mucosa was scraped from a 10–15 cm segment of jejunum. The content samples of duodenum, jejunum, ileum, and cecal were also collected. All samples were frozen in liquid nitrogen immediately and then stored at -70°C for further analysis.

### Enzyme Activity Analyses

Jejunal mucosa samples were homogenized in ice-cold 0.1 mol/L, pH = 6.8 maleic acid buffer (1:10, w/v) and centrifuged at 3000 ×*g* for 10 min. Supernatants were collected to determinate the activities of surcrase, maltase, lactuase, AKPase, Na^+^, K^+^-ATPase, γ-glutamyl transferase (γ-GT) and DAO, following the protocol of assay kit purchased from Nanjing Jiancheng Bioengineering Institute (Nanjing, China). The contents samples of duodenum, jejunum and ileum were homogenized with ice-cold physiologic saline (1:4, w/v) and supernatants were obtained by centrifugation at 3500 ×*g* for 15 min. The activities of chymotrypsin, amylase, trypsin, lipase in the supernatants of intestinal content were detected using the assay kit (Nanjing Jiancheng Bioengineering Institute, Nanjing, China).

### Western Blot Analysis

0.1 g jejunal mucosa was lysed with 500 μL cell lysis buffer for Western and IP (Beyotime Co. Ltd., Nantong, China). The lysates were centrifuged at 12,000 ×*g* for 5 min at 4°C and the supernatants were transferred to 1.5 ml Eppendorf tubes. The concentration of total protein was quantified by BCA Protein Assay Kit (Beyotime Co. Ltd., Nantong, China). Equal amount of cell lysates were resolved by SDS-PAGE, and then transferred electrophoretically to nitrocellulose membranes. After blocking with TBS containing 5% nonfat dry milk (Wondersun Inc., Haerbing, China) and 0.1% Tween-20 for 1 h at room temperature, the membranes were incubated with a primary antibody at 4°C overnight. After washing with TBST, membranes were incubated with secondary antibody linked to HRP. Detection by enzyme-linked chemiluminescence was performed follow the manufacturer’s protocol (ECL, Beyotime Co. Ltd., Nantong, China). Mouse anti-β-actin monoclonal antibody and IgG-HRP secondary antibodies were purchased from Beyotime Biotechnology (Nantong, China). Rabbit anti-SGLT1 and anti-PEPT1 were obtained from Abcam (MA, United States). Quantification of protein bands were analyzed using the Image J software.

### DNA Extraction and Illumina Miseq

Microbial genome DNA was extracted from fecal samples (using TIANamp Stool DNA kit; TIANGEN, DP328) following the manufacture’s recommendation. The V3-V4 hyper variable regions of 16S rRNA were PCR amplified from microbial genome DNA which were harvested from fecal samples (*n* = 3) forward primers: 5′-CCTACGGGNGGCWGCAG-3′, reverse primers: 5′-GACTACHVGGGTATCTAATCC-3′). A total volume of 20 μL was prepared, containing 1 × reaction buffer, 2 mM Mg^2+^, 0.2 mM dNTP, 0.1 μM primers, 1 U HotStarTaq polymerase (QIAGEN, cat#203203) and 2 μL DNA template. The PCR program initially started with 94°C for 2 min; 94°C for 20 s, 52°C for 40 s and 72°C for 1 min, 72°C for 2 min, repeat for 30 cycles; 72°C 2 min; stored in 4°C. The PCR reaction system which was used to add specific tags sequence was 20 μL, containing 1 × reaction buffer (NEB Q5TM), 0.3 mM dNTP, 0.25 M of each primer, 1 U Q5TM DNA polymerase (NEB) and l μL of diluted template. The PCR condition were 98°C for 30 s; 94°C for 10 s, 65°C for 30 s and 72°C for 30 s, repeat for 30 cycles; 72°C for 5 min. PCR product was excised from a 1.5% agarose gel, purified by QIAquick Gel Extraction Kit (QIAGEN, cat# 28706) and quantified by UV-Vis spectrophotometer (NanoDrop ND1000, United States). Library construction and Illumina MiSeq sequencing was carried out in G-Bio Biotech (Hangzhou) Co., Ltd. And the information of DNA sequences was analyzed by QIIME software (Caporaso et al., 2010).

### Statistical Analysis

The data in present study were analyzed by one-way ANOVA using the IBM SPSS 16.0. The values of *P* < 0.05 or 0.01were considered a statistically significant difference.

## Results

### Digestive Enzyme Activity in Intestinal Contents of the Piglets

**FIGURE 1:**
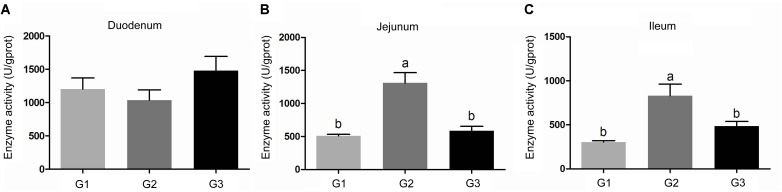
Effects of Ba on chymotrypsin activity in contents of duodenum **(A)**, jejunum **(B)**, and ileum **(C)**. Results were expressed as mean ± SEM, *n* = 6. a and b means significantly different (*p* < 0.05).

Compare with the G1 group, half replacing antibiotic with Ba significantly enhanced the activity of chymotrypsin (*P* < 0.05) in jejunum and ileum contents, while decreased the chymotrypsin activity in duodenum contents. The activities of chymotrypsin in different intestinal sections in G3 group were slightly increased in compare to the G1 group. It was much higher than that of the G2 group. However, in jejunum and ileum, they were dramatically lower when compared G2 group with G3 group (Figure 1).

**FIGURE 2:**
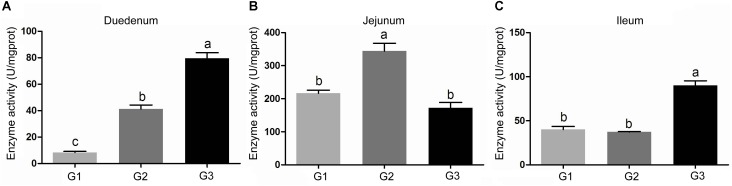
Effects of Ba on amylase activity in contents of duodenum **(A)**, jejunum **(B)**, and ileum **(C)**. Results were expressed as mean ± SEM, *n* = 6. a and b means significantly different (*p* < 0.05).

**FIGURE 3:**
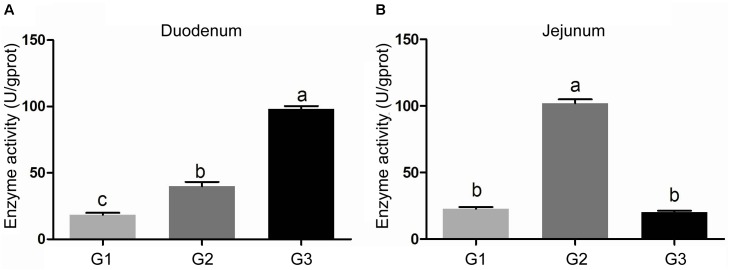
Effects of Ba on activity of lipase in contents of duodenum **(A)** and jejunum **(B)**. Results were expressed as mean ± SEM, *n* = 6. a and b means significantly different (*p* < 0.05).

**FIGURE 4:**
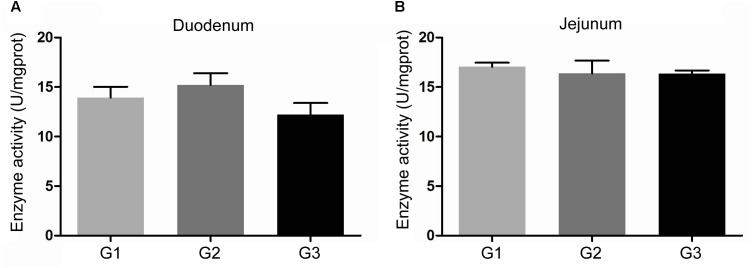
Effects of Ba on activity of trypsin in contents of duodenum **(A)** and jejunum **(B)**. Results were expressed as mean ± SEM, *n* = 6. a and b means significantly different (*p* < 0.05).

The activity of amylase in intestinal contents was shown in Figure 2. Half replacing antibiotic with Ba significantly enhanced the amylase activity (*P* < 0.05) in the jejuna content when compared with the G1 group, and the piglets in G3 group had the highest amylase activity (*P* < 0.05) in the contents of duodenum and ileum (*P* < 0.05) among the three groups. In addition, it was observed that half replacing the antibiotic with Ba significantly increased the lipase activity in duodenal and jejunal content compared with the G1 group, and that in G3 group was dramatically enhanced in comparison with the G2 group (Figure 3A), while there was dramatical difference between G1 and G3 group (Figure 3B). However, there were no significant changes of trypsin activity in the content of duodenum and jejunum (Figures 4A,B).

### Enzyme Activity Related to Absorption in Jejuna Mucosa of the Piglets

**FIGURE 5:**
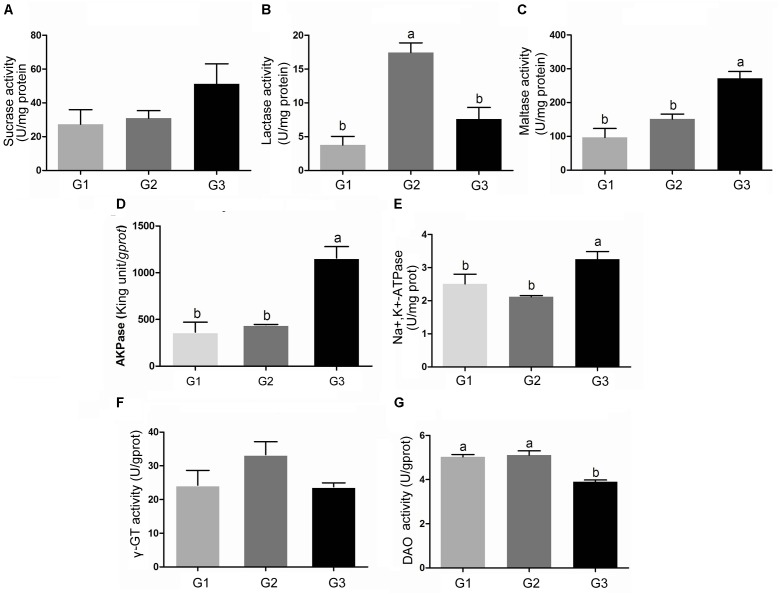
Effects of Ba on the activity of sucrase **(A)**, lactase **(B)**, maltase **(C)**, AKPase **(D)**, Na ^+^, K ^+^-ATPase **(E)**, γ-GT **(F)**, and DAO **(G)** in jejunal mucosa of piglets. Results were expressed as mean ± SEM, *n* = 6. a and b means significantly different (*p* < 0.05).

Figure 5 showed the results of sucrase (A), lactase (B), and maltase (C) activities. Half replacing the antibiotic with Ba only significantly increased the activity of lactase (*P* < 0.05) when compared with G1 group (Figure 5B). The piglets in G3 group had much higher activity of sucrase (*P* > 0.05), maltase (*P* < 0.05) compared with G1 and G2 group (Figures 5A,C). Compared with G1 group, half replacing the antibiotic with Ba did not affect the activity of AKPase, however when the piglet fed with Ba instead of antibiotic, AKPase activity was significantly improved (Figure 5D). And the same result was found in the activity of Na^+^, K^+^-ATPase (Figure 5E).

Feed supplemented with antibiotic or Ba did not induce any change in γ-GT activity in each group (Figure 5F). No significant difference of DAO activity was found between G1 and G2 group, while DAO activity in the piglets fed with Ba instead of antibiotic was significantly decreased (*P* < 0.05) (Figure 5G).

### Effects of Ba on Transporter Expression

**FIGURE 6:**
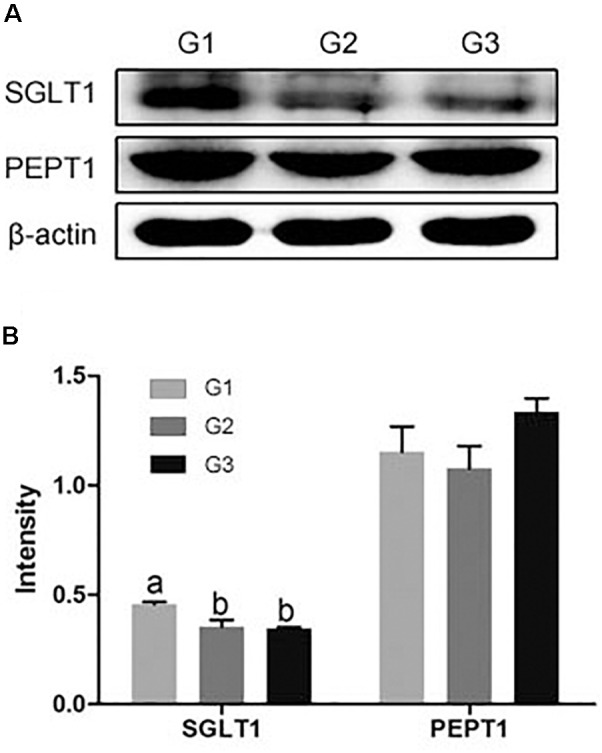
**(A)** Effects of Ba on the expression of SGLT1 and PEPT1 in jejuna mucosa of piglets. **(B)** Results were given as mean ± SEM, *n* = 6. a and b means significantly different (*p* < 0.05).

Compared with G1 group, half and total replacing the antibiotic with Ba both significantly reduced the *SGLT*1 expression in jejuna mucosa (*P* < 0.05), while no dramatical difference between G2 and G3 group (Figure 6). And half and total replacing the antibiotic with Ba did not affect the *PEPT*1 expression when compared with G1 group.

### Effects of Ba on Intestinal Microbiota

**FIGURE 7:**
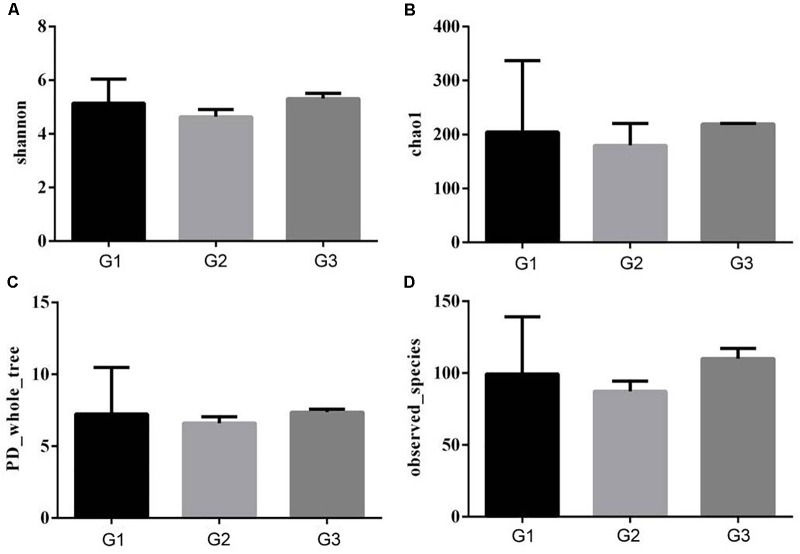
Effects of Ba on α-diversity of microbial population in piglets. Shannon **(A)**, Chao1 **(B)**, PD whole tree **(C)**, and observed species **(D)** was analyzed.

**FIGURE 8:**
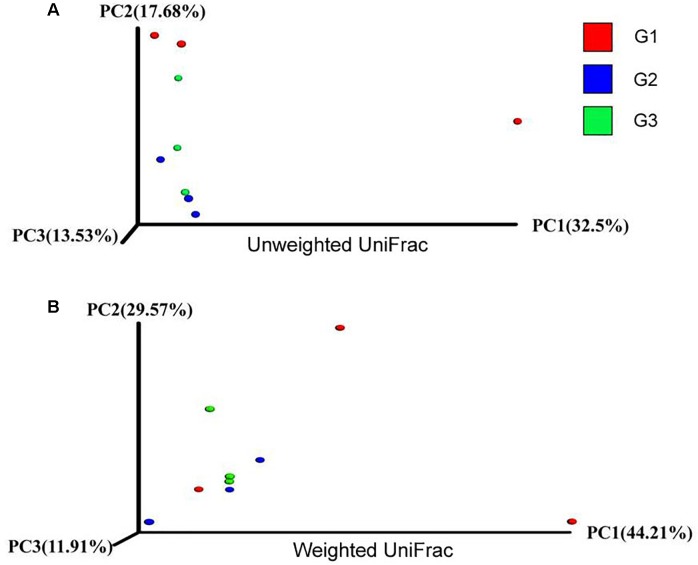
Effects of Ba on β-diversity of microbial population in piglets. Unweight unifrac **(A)** and weighted unifrac **(B)** was analyzed.

To further characterize the changes in microbial population imposed by the use of Ba and antibiotics, 16S rRNA were classified taxonomically to the genera level. Similar Shannon diversity indexes were found in G1–G3 group, as well as Chao1, PD whole tree and observed species (Figures 7A–D). To further measure the variability in species composition when the piglets were fed the diet with half and total replacing the antibiotic with Ba, β-diversity indexes were analyzed. Based on the unweighted UniFrac distance analysis, the difference between the intestinal flora of the intestinal flora of G1, G2, and G3 was significant (*p* = 0.028, Figure 8A), but based on the weighted UniFrac distance, no differences were found (*p* = 0.199, Figure 8B). While no significant changes were found among the different treatments in the phylum and genus level of gut bacteria (Supplementary Figures S1, S2 and Supplementary Tables S1, S2).

## Discussion

As more and more people are increasingly looking at food safety and environmental contamination, antibiotics replacement has become a trend. Probiotics have been widely used as antibiotic replacement to enhance animal growth and intestinal health (Caporaso et al., 2010; Deng et al., 2013). However, it remains unclear whether probiotics impact on the nutrients digestion and absorption of piglets. Previous study found that diet supplemented with *Bacillus amyloliquefaciens* partly instead of antibiotics dramatically improved the growth performance of piglets (Wang et al., 2017). Hence, our study focused on the influences of probiotic *Bacillus amyloliquefaciens* as an alternative of antibiotics on activities of digestive enzymes in piglet intestine.

Carbohydrates are one of the most important components of the diet. In digestive tract, carbohydrates are mainly digested by salivary and pancreatic amylases, further broken down into monosaccharides by disaccridase, such as sucrase, maltase and lactase, which secreted by the BBM of enterocytes, and then are absorbed (Drozdowski and Thomson, 2006; Zhen et al., 2018). Wang and Gu (2010) found that amylase activity was remarkably higher in Arbor Acres broilers feed with *Bacillus coagulans* NJ0516 than that in control group. In current study, amylase activity in duodenum and ileum significantly higher in piglets administrated with Ba than the piglets fed the diet supplemented with antibiotics or Ba half replacing antibiotics, and Ba half replacing antibiotics dramatically increased amylase activity in jejunum compared with the antibiotic group. Compared with the piglets fed the diet containing antibiotic, an increase in sucrase, maltase and lactase in jejuna mucosa was observed when they were fed the diet supplemented with Ba. And the same results were reported that when rats administrated with probiotics *Lactobacillus bulgaricus* and Streptococcus thermophilus, sucrase and lactase activity was enhanced in intestinal mucosa (Southcott et al., 2008), and Goyal et al. (2013) found that probiotic *Lactobacillus rhamnosus* GG dramatically increased the activity of sucrase and lactase in BALB/c mice. In addition, Na^+^/K^+^-ATPase is a transmembrane protein and is responsible for driving the sodium-dependent glucose transporter (SGLT1) in BBM, which plays an important role in glucose transport. It has shown that the inhibition of SGLT1 was secondary to a reduction in Na^+^/K^+^-ATPase activity (Manoharan et al., 2018). However, we observed that instead of antibiotic, feeding Ba-supplemented diet significantly increased the activity of Na^+^/K^+^-ATPase in jejuna mucosa, though the expression of SGLT1 was remarkably decreased in piglets fed with Ba. The results indicate that as an alternative of antibiotic, Ba could influence the metabolism of carbohydrates metabolism, while the certain further research was needed to clarify the certain effect of Ba.

Dietary protein is digested by both mammalian and bacterial enzymes in the intestinal tract (Goyal et al., 2013; Switzar et al., 2013). The protease activity was significantly higher in the common carp fed with basal diets supplemented with *Bacillus* sp. compared with control group (Wang and Xu, 2006). It has been suggested that in our study, compared to antibiotic fed group, activities of chymotrpsin was significantly increased in jejunual and ileal contents of piglets fed with Ba half replacing antibiotics. There was a tendency for increased activity of chymotrpsin when the piglet fed diet supplemented with Ba instead of antibiotic, while no remarkable changes were observed. However, activities of trypsin in intestinal contents and γ-glutamyl transpeptidase (γ-GT) in jejuna mucosa of piglets were not affected by the diet supplemented with Ba. And Ba treatment did not change the expression of peptide transporter 1 (PEPT1), which is a kind of membrane transporter proteins and helps the cellular of oligopepetides (Daniel, 2004).

In addition, the increase of lipase activity leads to more effective fat absorption (Lowe, 1994; Karásková et al., 2015). However, Ogawa et al. (2015) indicated that *Lactobacillus gasseri* SBT2055 significantly decreased the lipase activity in order to increase size of fat emulsion droplet and suppress lipid absorption. In current study, we found that piglets in replacing half dosage of antibiotic with Ba had much higher activity of lipase in contents of duodenum and jejunum than piglets fed with antibiotic (*P* < 0.05), and piglets in Ba groups had the highest lipase activity in duodenum, although in jejunum, lipase activity was significantly decreased in piglets fed with Ba compared to Ba half replacing antibiotics group.

The intestinal environment plays a critical role in maintaining good health (Farhadi et al., 2003; Melo et al., 2015). DAO is one of the indicators of intestinal epithelial integrity (Tossou et al., 2016). It has been reported that antibiotic treatment significantly lowered DAO activity (Sato et al., 2016). However, in the present study, piglets administrated with Ba had much lower DAO activity compared with antibiotic group, which may indicate much better state of intestinal integrity. AKPase is a new factor which contributes to maintain gut homeostasis (Lalles, 2014; Melo et al., 2015). The deletion of intestinal AKPase gene caused a significant decrease of tight junction protein expression and function (Liu et al., 2016). In the present study, we found that Ba replacing antibiotic dramatically enhanced the activity of AKPase.

Intestinal microbiota is an important compartment of digestive tract of animals (Azad et al., 2018). Various researches have demonstrated that probiotic can positively regulate the composition of the intestinal macobiota (Cisek and Binek, 2014; Kristensen et al., 2016; Hu et al., 2017). Dietary supplementation of Bacillus amyloliquefaciens dramatically decreased the population of Escherichia coli and increased Lactobacillus population in cecum (Lei et al., 2014). The administration of Lactobacillus B1 significantly decreased the number of E. coli and increased lactic acid bacteria in cecal digesta of chickens (Peng et al., 2016). While in the current study, no significant influences were found when the piglet fed with Ba instead of antibiotics.

## Conclusion

In conclusion, results from the previous study indicate that supplementation with Ba enhanced growth performance of piglets (Wang et al., 2017). This enhancement was associated with the positive influence of tract digestibility and intestinal integrity. Therefore, according to our research, *Bacillus amyloliquefaciens* SC06 could be used as alternative to antibiotics in piglet diets.

## Author Contributions

WL and LG designed the experiment. SH and XC drafted the manuscript. XC carried out most of the experiments and analyzed the data. YWu, XM, and HX participated in the animal experiment. YWa helped to detect some parameters. XZ performed the analysis of the microbiota. WL and LG had primary responsibility for the final content.

## Conflict of Interest Statement

The authors declare that the research was conducted in the absence of any commercial or financial relationships that could be construed as a potential conflict of interest.

## Supplementary Material

FIGURE S1 The changes of gut bacterial phyla in different treatments.

FIGURE S2 The changes of gut bacterial genera in different treatments.

TABLE S1 Detailed data for the gut bacterial phyla.

TABLE S2 Detailed data for the gut bacterial genera.

## Abbreviations

γ-GT: γ-glutamyltranspeptidase
AKPase: Alkaline phosphatase
BBM: Brush border membrane
DAO: Diamine oxidase
PEPT1: Peptide transporter 1
SGLT1: Sodium-dependent glucose transporter
