# Supplementary figures and images for: Effects of Probiotic Bacillus as an Alternative of Antibiotics on Digestive Enzymes Activity and Intestinal Integrity of Piglets
Source: Front Microbiol. 2018 Oct 22;9:2427. doi: 10.3389/fmicb.2018.02427 (PMC6204369; doi:10.3389/fmicb.2018.02427)

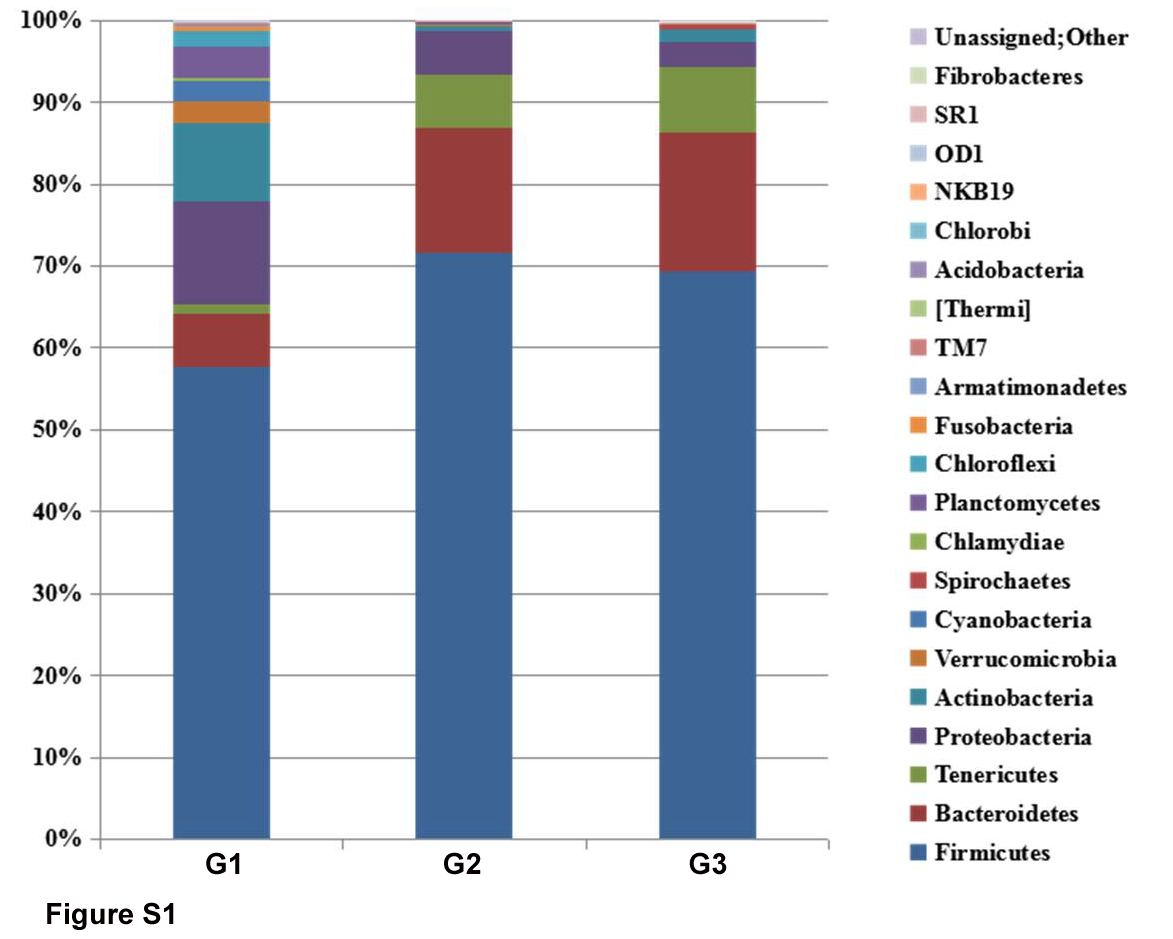

Supplement: FIGURE S1 — The changes of gut bacterial phyla in different treatments. [file Image_1.JPEG]

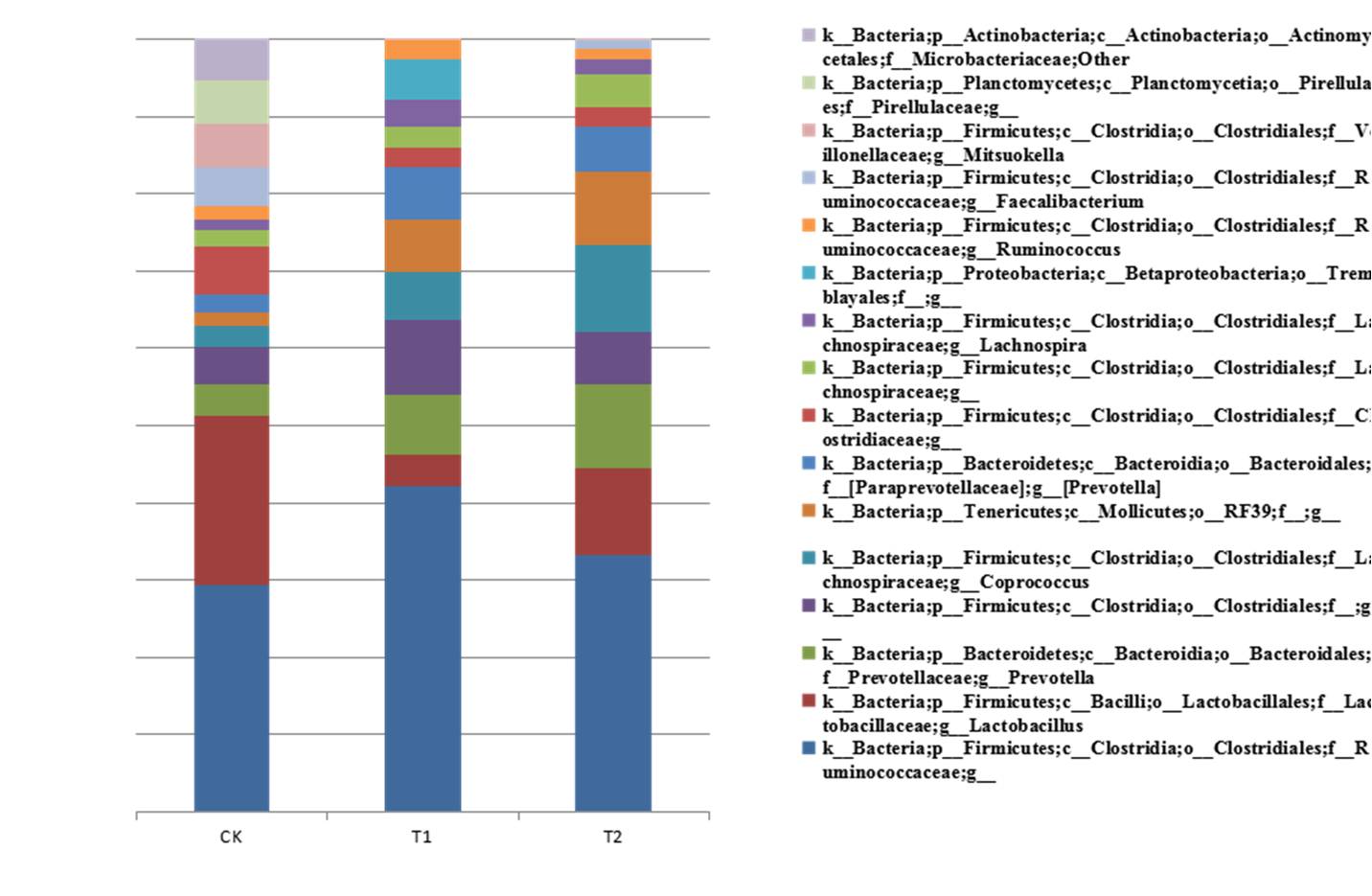

Supplement: FIGURE S2 — The changes of gut bacterial genera in different treatments. [file Image_2.JPEG]
